# Adaptive Accumulation of Plantar Pressure for Ambulatory Activity Recognition and Pedestrian Identification

**DOI:** 10.3390/s21113842

**Published:** 2021-06-02

**Authors:** Phuc Huu Truong, Sujeong You, Sang-Hoon Ji, Gu-Min Jeong

**Affiliations:** 1 Robot Division, Korea Institute of Industrial Technology, Ansan 15588, Korea; phtruong@kitech.re.kr (P.H.T.); sjyou21@kitech.re.kr (S.Y.); robot91@kitech.re.kr (S.-H.J.); 2 School of Electrical Engineering, Kookmin University, Seoul 02707, Korea

**Keywords:** ambulatory activity recognition, pedestrian identification, plantar pressure, smart shoes, gait monitoring

## Abstract

In this paper, we propose a novel method for ambulatory activity recognition and pedestrian identification based on temporally adaptive weighting accumulation-based features extracted from categorical plantar pressure. The method relies on three pressure-related features, which are calculated by accumulating the pressure of the standing foot in each step over three different temporal weighting forms. In addition, we consider a feature reflecting the pressure variation. These four features characterize the standing posture in a step by differently weighting step pressure data over time. We use these features to analyze the standing foot during walking and then recognize ambulatory activities and identify pedestrians based on multilayer multiclass support vector machine classifiers. Experimental results show that the proposed method achieves 97% accuracy for the two tasks when analyzing eight consecutive steps. For faster processing, the method reaches 89.9% and 91.3% accuracy for ambulatory activity recognition and pedestrian identification considering two consecutive steps, respectively, whereas the accuracy drops to 83.3% and 82.3% when considering one step for the respective tasks. Comparative results demonstrated the high performance of the proposed method regarding accuracy and temporal sensitivity.

## 1. Introduction

Ambulatory activity analysis is fundamental for the study of human movement because it can provide rich information for gait analysis [1,2,3]. In addition, ambulatory activity analysis is an important tool for fall detection and post-stroke rehabilitation and, thus, can contribute to enhancing the quality of life, especially for the elder population [4,5]. Therefore, many wearable sensor-based approaches have been proposed for analyzing ambulatory activity [6,7,8,9].

Various methods for ambulatory activity analysis rely on inertial sensors such as accelerometers and gyroscopes to study body motion. Karantonis et al. [7] developed a classification system for human movements using a triaxial accelerometer attached to the waist of subjects. Similarly, Trost et al. [8] analyzed the accuracy of attaching a triaxial accelerometer to hip and/or wrist for ambulatory activity recognition. The results showed that attaching a sensor to the hip provides better accuracy than attaching it to the wrist. In [9], the authors combined inertial sensors with a barometer sensor and attached the sensors to subjects’ wrists, ankles, and trunks to improve activity recognition.

Alternatively, ambulatory activity can be analyzed using data from a pressure sensor placed under the subjects’ feet. These data reflect the spatial and temporal distribution of foot force [10]. Moreover, pressure sensors accurately indicate static activities (e.g., standing and sitting) and motion activities from the pressure variance, whereas inertial sensors can only describe motion activities. Several studies using pressure sensors for ambulatory activity analysis have been conducted. Zhang et al. [11] used foot-force sensors to recognize transportation activities by adopting statistical features of foot pressure and the random forest algorithm to classify the activities. Jeong et al. [12] have proposed a method for ambulatory activity recognition based on plantar pressure, achieving 95.2% accuracy when considering six walking steps. Pappas et al. [13] placed a gyroscope and three force-sensitive resistors on a shoe sole to detect the gait phases. Similarly, by combining an insole endowed with pressure sensing and two six-axis inertial measurement units, Mazumder et al. [3] accurately classified ambulatory activities.

Recently, deep learning methods have been applied to overcome various challenges for sensor-based activity recognition [14]. Multiple long short-term memory (LSTM) cells have been stacked to recognize human activity from inertial data [15]. Zhao et al. [16] combined a residual module with LSTM to create a network that enhances the recognition rate. A convolutional neural network (CNN) has also been stacked with LSTM for human activity recognition [17].

Ambulatory activity analysis also enables person identification. In fact, the analysis of people’s walking patterns using wearable sensors can lead to individual identity [18,19]. In [18], Huang et al. combined a CNN-based network with the extended Kalman filter (EKF) algorithm to identify each person, using data from force and inertial sensors attached to shoes. In [19], plantar pressure and inertial data were separately processed using CNN and LSTM-based networks, and the outcomes were fused for pedestrian identification.

In this paper, we explore useful features to analyze ambulatory activity and achieve activity recognition and pedestrian identification using insole pressure sensors. Specifically, we propose a novel method of recognizing three ambulatory activities (i.e., level walking, stair descent and stair ascent) and identifying 29 pedestrians using plantar pressure data. In addition, we introduce three handcrafted pressure features, called temporally adaptive weighting accumulation (TAWA), to efficiently analyze walking characteristics. Feeding these features into two multilayer, multiclass support vector machine (SVM) classifiers, one classifier for ambulatory activity recognition and another one for pedestrian identification, and training in the supervised protocol, we obtain accuracies higher than 97% for both activity recognition and pedestrian identification tasks when analyzing eight consecutive steps. When considering two consecutive steps, the accuracies achieve 89.87% and 91.34%, and f1-scores achieve 89.72% and 91.91% for activity recognition and pedestrian identification, respectively. The proposed method substantially outperforms similar methods regarding both activity recognition and pedestrian identification tasks.

## 2. Problem Definition

We aim to obtain handcrafted features for accurately recognizing ambulatory activities and identifying pedestrians using plantar pressure measurements. The plantar pressure data are measured in sequence using pressure sensors of smart insoles placed under pedestrians’ shoes. The representative features should well-describe the ambulatory activities for accurate activity recognition and pedestrian recognition. The problem addressed in this study can thus be detailed as follows:

*Given*:

- Sequences of plantar pressure data from insole;
- Corresponding ambulatory activities and the participant performing each activity.

*Objectives*:

- Design a parametric model for activity recognition;
- Design a parametric model for pedestrian identification.

## 3. Methodology

### 3.1. Study Protocol

The paper aims to recognize three ambulatory activities; namely, level walking, stair descending and stair ascending, performed by healthy participants, and then identifying these subjects using plantar pressure data obtained from smart insoles placed under their shoes. The participants do not have any walking disorders. Specifically, twenty-nine healthy university students (23 males and 6 females) were recruited in this study to perform the three ambulatory activities. These students did not receive any treatment for gait disorder during experimentation. The participants were informed of the purpose of the data collection and agreed to participate in the study. The participants signed the consent letter to confirm their participation. The participants reserve the right to refuse collection or storing the data.

In the experiments, each participant stood on a starting mark of the walking distance in each experiment type (level walking, stair descending and stair ascending) in turn and waited for our instruction to begin a trial. After indicating the participant to start, the subject triggered the data collection function on a mobile application by pushing the corresponding button on the screen of a smartphone. Then, the participant performed each activity. After 10–12 steps of walking, the participant stopped the activity and then halted the data collection. The same protocol was adopted for the three activities. The participants were required to complete from 12 to 20 trials per activity. The phone received the data recorded from the insoles through Bluetooth connection and stored them in separate files for each trial. Using this protocol, the collected data are separated for each activity and each participant without any manual annotation.

### 3.2. Measurement System

**Figure 1:**
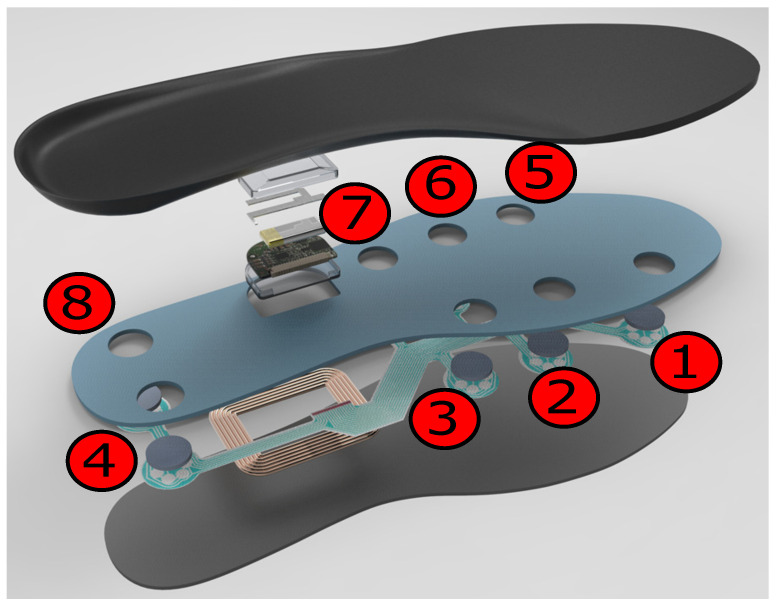
Smart insole to acquire plantar pressure data. Numbers 1–8 represent the positions of eight sensor nodes which measure the force exerted on the insole.

To collect plantar pressure data, we developed a pressure monitoring system that wirelessly connects a smartphone with two smart insole sensors [12]. Each insole sensor contains eight pressure sensors that periodically measure the force exerted on them. The pressure sensors are distributed on the insole’s area. The insoles provide plantar pressures in four discrete intensive levels {0, 1, 2, 3} corresponding to “no pressure”, “slight pressure”, “moderate pressure” and “high pressure”. The composition of the smart insole with its eight pressure sensors is shown in Figure 1.

We developed a mobile application to seamlessly acquire pressure data from each sensor in the smart insoles through a Bluetooth interface. In the application, a thread is constructed and started on a smartphone to periodically obtain pressure data from the two smart insoles. Using this acquisition system, we recorded the plantar pressure under the two feet of each participant while they were performing three ambulatory activities. The participants were required to hold the smartphone in their hand and place the two insoles in their shoes during the experiment. The pressure is recorded at the frequency of 50 Hz.

### 3.3. Problem Solution

Consider an input dataset $X=\left\{x_{1},x_{2},\cdots ,x_{N}\right\}$ with $x_{k}={\left[x_{1},x_{2},\cdots ,x_{d}\right]}^{T}$ corresponding to a target set $Y=y_{1},y_{2},\cdots ,y_{k}$ with $y_{k}\in \left\{1,2,\cdots ,c\right\}$, of *c* predefined classes (i.e., the number of activities or the number of subjects to be recognized). The classification problem can be defined as the construction of a model *f* that is governed by the vector w of adaptive parameters so that *f* can best map each input vector $x_{k}$ onto each label $y_{k}$ of the label set of predefined classes.

By applying the Bayes rule, the posterior probability of the weights can be expressed as

(1) $$p\left(w|X\right)=\frac{p(X|w)p(w)}{p(X)}.$$

Assume that the prior p(w) is a zero-mean Gaussian function of weights, such that:

(2) $$p\left(w|X\right)\propto exp\left(-\frac{\alpha }{2}\sum_{w}{∥w∥}^{2}\right),$$

the likelihood of the model can be written as

(3) $$p\left(X|w\right)=p\left({\left\{y_{i}\right\}}_{i=1}^{N}|{\left\{f(x_{i})\right\}}_{i=1}^{N}\right).$$

We assume that the model is drawn independently, and thus the $y_{i}$ are conditionally independent from $f(x_{i})$, and, the likelihood is multiplicative. Following the assumption of multiclass SVM [20,21], we use the following output probability:

(4) $$p\left(y_{i}|f\left(x_{i}\right)\right)\propto exp\left\{-\sum_{j=1,j\neq y_{i}}^{c}{\left(f_{j}\left(x_{i}\right)+\frac{1}{\psi -1}\right)}_{+}\right\},$$

under the constrain $\sum_{j=1}^{c}f_{j}\left(x\right)=0$, where ${\left(u\right)}_{+}=u$ if u>0 and ${\left(u\right)}_{+}=0$ otherwise, whereas ψ is a marginal constant, such that:

(5) $$\begin{array}{cc} p(X|w) & =\prod_{i=1}^{N}exp\left\{-\sum_{j=1,j\neq y_{i}}^{c}{\left(f_{j}\left(x_{i}\right)+\frac{1}{\psi -1}\right)}_{+}\right\}\\ \; & =\prod_{i=1}^{N}exp\left\{-\sum_{x_{i}\notin X_{j}}{\left(f_{j}\left(x_{i}\right)+\frac{1}{\psi -1}\right)}_{+}\right\}. \end{array}$$

where, $X_{j}$ represents the input set belonging to the *j*-th class. Probability p(X) can be ignored in the Bayesian, because it can be interpreted as the normalization of the posterior probability. Thus, the posterior can be written as

(6) p(w|X)∝p(X|w).p(w).

Maximizing the logarithm of the posterior, we obtain the following optimization problem:

(7) $$\begin{array}{cc} w & =\underset{w}{arg max}\left(log(p(w|X)\right)\\ \; & =\underset{w}{arg max}\left(-\sum_{i=1}^{N}\sum_{x_{i}\notin X_{j}}{\left(f_{j}\left(x_{i}\right)+\frac{1}{\psi -1}\right)}_{+}-\frac{\alpha }{2}\sum_{w}{∥w∥}^{2}\right)\\ \; & =\underset{w}{arg min}\left(\sum_{i=1}^{N}\sum_{x_{i}\notin X_{j}}{\left(f_{j}\left(x_{i}\right)+\frac{1}{\psi -1}\right)}_{+}+\frac{\alpha }{2}\sum_{w}{∥w∥}^{2}\right)\\ s.t.\\ \; & \sum_{j=1}^{c}f_{j}\left(x\right)=0. \end{array}$$

### 3.4. TAWA-Based Activity Recognition and Pedestrian Identification

**Figure 2:**
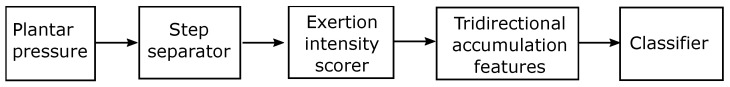
TAWA-based method of activity recognition and pedestrian identification.

Using the data obtained from the measurement system, we first perform step detection. Based on the results of the step detection, the plantar pressure data are segmented into steps and further processed for activity recognition. Figure 2 depicts the proposed method’s architecture. We aim to accurately recognize the activity using the minimum number of walking steps for fast performance.

Applying our step-detection method [22], we detect steps, determine swing and stance phases, and segment the pressure data from both feet per step and phase. In this paper, we propose a technique to extract important features from time-series pressure data in a step $p\left(t\right)=[p_{i}\left[0\right],p_{i}\left[1\right],\cdots ,p_{i}\left[t\right]]$. This technique assigns weights to each pressure component in the stance phase in incremental, decremental and equal contributions and then provides the accumulation in these three types of contributions to form a TAWA feature set $z={\left[z_{1},z_{2},\cdots ,z_{d}\right]}^{T}$. The feature set is used for activity recognition and pedestrian identification. The features are fed into a perceptron layer with rectified linear unit (RELU) activation and then a linear scorer to compute the sample scores for activity recognition. A similar structure is applied to compute the sample scores for pedestrian identification. Let $g\left(x\right)=w_{\left(1\right)}^{T}x+b_{\left(1\right)}$ and $h\left(x\right)=w_{\left(2\right)}^{T}x+b_{\left(2\right)}$ be functions governed by adaptive parameters at the perceptron and RELU layers, the samples scores can be expressed as:

(8) $$f\left(p_{i}\right)=h\left(g{\left(z_{i}\right)}_{+}\right)=w_{\left(2\right)}{\left(w_{\left(1\right)}z_{i}+b_{\left(1\right)}\right)}_{+}+b_{\left(2\right)}$$

From score $f(p_{i})$, we find the optimal parameter set w according to Equation (7) by applying the stochastic gradient descent method.

### 3.5. Temporally Adaptive Pressure Accumulation

**Figure 3:**
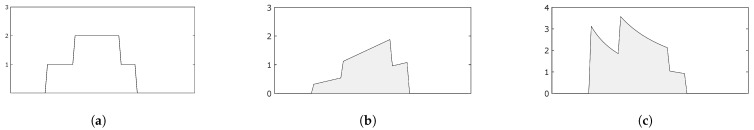
Accumulation of plantar pressure data from a sensor per step. (**a**) Plantar pressure of a sensor in one step. (**b**) Temporally increasing weighting accumulation. (**c**) Temporally decreasing weighting accumulation.

Feature extraction using TAWA of pressure data aims to calculate the pressure accumulation per step in three ways: temporal increase, temporal decrease and temporal independent accumulation. The temporal increase and decrease assign higher weights to the pressure data in the late and early stages of a step, respectively, whereas the temporally independent accumulation equally weights the pressure data from a step. Figure 3 illustrates the temporal increase and decrease accumulation of the pressure data per step. Figure 3a shows a sample of the pressure data recorded by one sensor in a step. The temporally increasing and decreasing weighting accumulation features are defined by the gray areas in Figure 3b,c, respectively.

The temporally increasing weighting accumulation for each of the eight sensors is calculated as follows:

(9) $$\begin{array}{c} f_{1}^{\left(i\right)}=\frac{1}{T}\int_{t=0}^{T}P_{i}\left(t\right)\times tdt, \end{array}$$

(10) $$\begin{array}{c} f_{1}^{\left(i\right)}=\frac{1}{T}\sum_{k=\Delta T}^{T}P_{i}\left[k\right]\times k, \end{array}$$

where, $P_{i}\left(t\right)$ is the pressure collected from the *i*-th sensor at time step *t* within step interval [0,T]. The contribution of pressure data within a swing phase is determined according to their time step in the swing phase. Hence, the temporal increase emphasizes the late stage of the stance phase in the walking cycle. This feature reflects the characteristics of the pre-swing stage in a walking cycle. In contradiction, the temporal decrease emphasizes the early pressure data during accumulation. This feature tracks the pressure during the early stance phase (i.e., initial contact stage) in the walking cycle:

(11) $$\begin{array}{c} f_{2}^{\left(i\right)}=\frac{1}{T}\int_{t=0^{+}}^{T}\frac{P_{i}\left(t\right)}{t}dt, \end{array}$$

(12) $$\begin{array}{c} f_{2}^{\left(i\right)}=\frac{1}{T}\sum_{k=\Delta T}^{T}\frac{P_{i}\left[k\right]}{k}. \end{array}$$

Next, the time-independent feature integrates the pressure data in a step by equally weighting the pressure distribution. The temporally independent weighting accumulation is defined by the area of the pressure in a step, as shown in Figure 3a. This feature can be computed as:

(13) $$\begin{array}{c} f_{3}^{\left(i\right)}=\frac{1}{T}\int_{t=0}^{T}P_{i}\left(t\right)dt, \end{array}$$

(14) $$\begin{array}{c} f_{3}^{\left(i\right)}=\frac{1}{T}\sum_{k=\Delta T}^{T}P_{i}\left[k\right]. \end{array}$$

**Figure 4:**
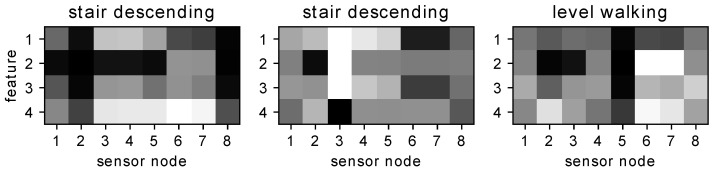
Maps of features calculated from different sensor nodes in a step.

Since this time-independent feature is a scaled version of the mean of the pressure data per step, it characterizes the general plantar pressure distribution on each sensor during a step that is high in the stance phase and low in the strike and toe-off stages. For activity recognition and pedestrian identification, the variation of the pressure in the walking cycle should also be characterized. Thus, we include pressure variation feature $f_{v}^{\left(i\right)}$, which is calculated as the standard deviation of the pressure data in a step for recognition. After normalization, these four features are fed into the multiclass SVM classifiers to determine the type of activity and identify the pedestrian. Figure 4 visualizes the four features in a step of three activities. The vertical axis represents the TIWA, TDWA, TIWA and STD, respectively, whereas the horizontal axis represents the sensors of the insole with respect to Figure 1.

## 4. Experimental Results

Using the developed system, we collected data from 1207 trials of three walking activities (i.e., level walk, stair descent and ascent) from the 29 participants. Each trial corresponds to a participant performing one of the three ambulatory activities over a predefined distance of 12 to 16 walking steps. We organized the trials into a dataset for offline evaluation. The dataset contains the samples from 17150 steps of three ambulatory activities recorded from the 29 participants. To evaluate recognition, we split the dataset into training and test sets using 5-fold cross-validation. Furthermore, we evaluate the activity recognition and pedestrian identification of each method for different numbers of consecutive steps. To consider a various number of steps, we applied a sliding window to each sequence of the pressure data and split the samples for various numbers of steps. Table 1 lists the number of generated samples according to the number of steps used for activity recognition and pedestrian identification.

### 4.1. Ambulatory Activity Recognition

Applying the proposed method to the dataset, we obtained the activity recognition results listed in Table 2 according to the number of steps used for classification. The results are obtained by the use of TAWA features and their combination with the standard deviation (STD) feature. The number of steps represent consecutive steps in a walking trial.

**Figure 5:**
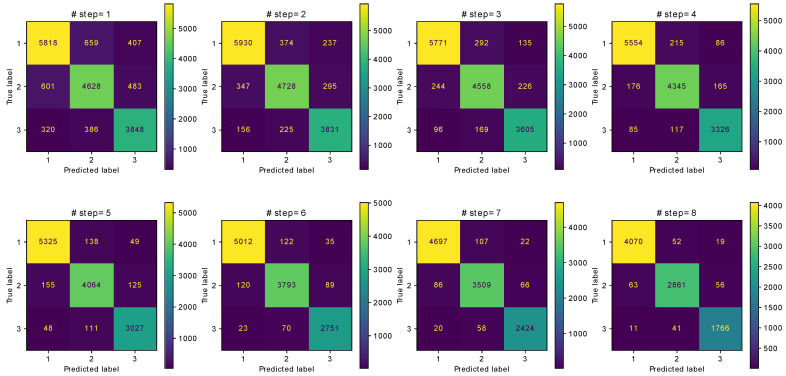
Confusion matrices of ambulatory recognition with respect to the number of steps.

Figure 5 provides the confusion matrices of activity recognition according to the number of consecutive steps using the combination of TAWA and STD features. The activities (stair descending, stair ascending and level walking) are represented by the numbers (1, 2 and 3), respectively. Each confusion matrix is calculated for all 5-fold cross-validation. In other words, we group all the labels and predictions for all 5-fold cross-validation to compute the final confusion matrix for each number of consecutive steps.

The classification accuracy increases as more steps are considered because more information becomes available for feature extraction. Thus, considering more steps increases the accuracy of the proposed method. Although the accuracy sharply increases up to four steps, it increases gradually slower when further adding steps, indicating convergence of the classifier. Using only TAWA features, the highest accuracy is 97.24% for eight steps. Adding the standard deviation feature, the accuracy further increases to 97.29% for eight steps. In addition, the proposed method provides accuracy and F1 score above 83% using the plantar pressure data from a single step. The F1 score values are comparable to the accuracy values, indicating that the dataset is balanced.

From the experiments, we observed that the average walking speed is approximately 1.2 m/s, and each step is approximately 0.6 m on average. Thus, accurate identification considering eight steps would take 4 s, which may be considered as a long period, and recognition requiring more than eight steps may be considered to be slow. We evaluate the method for one step and one stride (i.e., two steps) and show the recognition results in Table 3. The recognition would take approximately 0.5 and 1 s, respectively. Because the sampling frequency is 50 Hz, these intervals correspond to 25 and 50 samples, respectively.

To demonstrate the superior performance of the proposed method, we compared it with the FF [11], PPAC [12] methods and LSTM-based [15] methods. The FF method uses (31) statistical features from insole pressures of both feet and applies a C4.5 decision tree algorithm for classification. This method requires at least two steps for recognition. The PPAC method only uses the temporally independent accumulation features. The LSTM-based method is based on two stacked LSTM cells, a fully connected layer, and a softmax activation to calculate the probability of each activity. Table 3 lists the comparison results.

The proposed method provides the best recognition results on all measures when considering either one or two steps. Note that the proposed method considering only one step notably outperforms the other reference methods regarding accuracy, precision, recall, and F-measure. Therefore, the extracted plantar pressure features are effective for recognizing the three fundamental ambulatory activities in terms of accuracy and speed.

### 4.2. Pedestrian Identification

We also evaluated the proposed method for pedestrian identification, aiming to recognize the participant who was performing the activity. The number of classes for pedestrian identification is much higher than for ambulatory activity recognition (29 vs. 3). To demonstrate the generalization ability of the proposed method to classify a varying number of classes, we use the same-structure classifiers for both tasks. Training for pedestrian identification was conducted using the same protocol and settings used for activity recognition. Table 4 lists the results of pedestrian identification.

**Figure 6:**
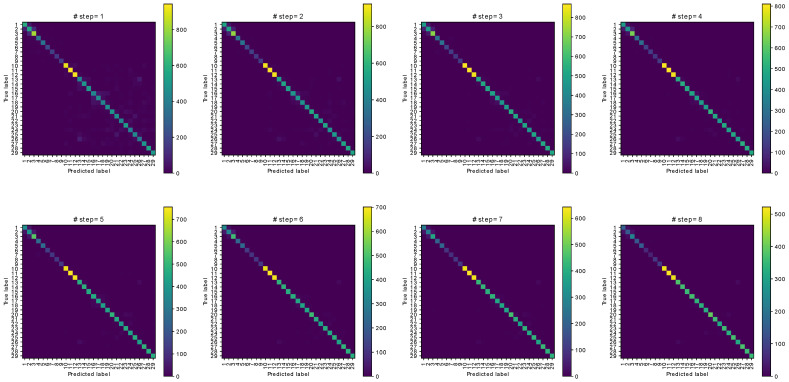
Confusion matrices of pedestrian identification with respect to the number of steps.

Figure 6 provides the confusion matrices of pedestrian identification according to the number of consecutive steps using the combination of TAWA and STD features. The participants are represented by their ID numbers. Because the number of participants is too large to display, we do not show the number of each element of the confusion matrices. Instead, we visualize the values by coloring them according to the referenced color bar on the right of each subfigure.

Similar to activity recognition, the accuracy and F1 score for pedestrian identification increase with the number of considered steps. Compared with the results of activity recognition, the accuracy and F1 score are similar for pedestrian identification. For eight consecutive steps, we obtain 96.04% accuracy and 96.57% for the F1 score, which are approximate to the corresponding values for activity recognition (96.64% and 97.10%). Therefore, the proposed method can be used for classification with a varying number of target classes. We also compared the proposed method with similar methods for pedestrian identification, obtaining the results listed in Table 5.

The proposed method substantially outperforms the comparison methods in all the measures when considering one and two steps. The performance of the proposed method using one step is notably higher than that of the other methods. Hence, the proposed method provides high recognition accuracy and temporal sensitivity, and the proposed features extracted from categorical plantar pressure are useful to analyze the three ambulatory activities and identify the pedestrian performing the activity.

## 5. Discussion

The proposed plantar pressure features enable the effective recognition of three activities and the identification of 29 pedestrians because they not only consider pressure data equally over one step but also emphasize on the early, late stages of the pressure distribution under each sensor of the insoles. The temporally increasing weighting accumulation feature emphasizes pressure data in the late stage of a step because it increasingly weights each data sample as the step proceeds. On the other hand, the temporally decreasing weighting accumulation feature emphasizes pressure data in the early stage of a step, and the temporally independent weighting feature equally weights pressure data in a step.

The temporally independent weighting feature corresponds to the expectation over time of plantar pressure in a step. Thus, it indicates the pressure intensity of the foot on the shoe. As the pressure intensity reflects the force employed to perform ambulatory activities individually, it plays an important role in ambulatory activity recognition and pedestrian identification.

From Equation (10), if we normalize the pressure data per step for the summation of data to be one, the temporally increasing weighting accumulation feature is the product of the expectation of the pressure moment with the total pressure in a step. This transformation can be expressed as $f_{tiwa}^{i}=\sum_{t}\left(P_{i}\left[t\right]\times t\right)=S_{P}^{i}\sum_{t}\left(p^{i}\left[t\right]\times t\right)$ with $S_{P}^{i}=\sum P^{i}\left[t\right]$ and $\sum p^{i}\left[t\right]=1$. If we consider $p^{i}\left[t\right]$ as the probability of t and ignore the total pressure in a step, the summation is the expectation of the pressure moment E[t] of the standing foot within a step. Thus, the temporal increasing feature characterizes the plantar pressure on the standing foot in a step and the duration of the step recorded by each pressure sensor.

Similarly, the temporally decreasing feature can be written as $f_{tdwa}^{i}=S_{P}^{i}E\left[\frac{1}{t}\right]$. Hence, this feature is the product of the total pressure and expectation of the pressed frequency and characterizes the overall pressure on the standing foot and the frequency of this pressure in a step. Combining these features, we can accurately recognize ambulatory activities and identify the pedestrian performing the activity.

## 6. Conclusions

We proposed a novel feature extraction-based method for classifying three ambulatory activities and identifying 29 pedestrians using only categorical plantar pressure data. The proposed method is based on new handcrafted features that generalize and extend existing features [12] extracted from plantar pressure in a step. Analyzing the existing feature, we proposed two new features for ambulatory activity analysis. We detailed and used these features as a new group of plantar pressure information. We also proposed extended multilayer, multiclass SVM classifiers for activity recognition and pedestrian identification. Applying the extracted feature, the proposed method substantially outperforms similar methods regarding accuracy and speed for the recognition of the three ambulatory activities and the identification of 29 pedestrians. The experimental results demonstrate the applicability and high performance of the proposed method.

## Figures and Tables

**Table 1:** Number of samples available according to the number of steps.

No. Steps	1	2	3	4	5	6	7	8
No. Samples	17,150	16,123	15,096	14,069	13,042	12,015	10,989	9963

**Table 2:** Activity recognition results with respect to step number.

No. Steps	1	2	3	4	5	6	7	8
Only TAWA								
Accuracy	82.43%	89.11%	91.67%	93.74%	95.10%	96.18%	96.33%	97.24%
F1 score	82.25%	88.95%	91.54%	93.63%	94.98%	96.08%	96.25%	96.71%
TAWA + STD								
Accuracy	83.35%	89.87%	92.30%	94.00%	95.20%	96.18%	96.73%	97.29%
F1 score	83.18%	89.72%	92.17%	93.87%	95.05%	96.09%	96.64%	97.07%

**Table 3:** Performance in each activity.

Method	Activity	Accuracy	Precision	Recall	F-Measure
	Level walk		65.53%	55.98%	60.38%
FF [11]	Stair descent		57.66%	60.53%	59.06%
(2 steps)	Stair ascent		62.93%	69.01%	65.83%
	Mean	61.83%	62.04%	61.84%	61.76%
	Level walk		87.04%	85.49%	86.26%
PPAC [12]	Stair descent		78.74%	79.89%	79.31%
(2 steps)	Stair ascent		81.46%	82.19%	81.82%
	Mean	82.76%	82.41%	82.52%	82.46%
	Level walk		92.51%	91.80%	92.15%
Proposed	Stair descent		88.86%	88.96%	88.91%
(2 steps)	Stair ascent		89.64%	90.59%	90.12%
	Mean	89.87%	89.58%	89.89%	89.72%
	Level walk		78.09%	78.36%	78.22%
PPAC [12]	Stair descent		70.72%	69.56%	70.13%
(1 step)	Stair ascent		71.65%	72.77%	72.21%
	Mean	73.94%	73.49%	73.56%	73.52%
	Level walk		81.34%	83.48%	82.40%
LSTM [15]	Stair descent		78.31%	77.27%	77.79%
(1 step)	Stair ascent		78.84%	77.10%	77.96%
	Mean	79.69%	79.50%	79.28%	79.38%
	Level walk		85.60%	86.20%	85.90%
Proposed	Stair descent		83.05%	79.62%	81.30%
(1 step)	Stair ascent		81.44%	84.80%	83.09%
	Mean	83.35%	83.04%	83.34%	83.18%

**Table 4:** Pedestrian identification results with respect to step number.

No. Steps	1	2	3	4	5	6	7	8
Only TAWA								
Accuracy	81.42%	90.76%	92.85%	94.33%	95.18%	95.79%	96.17%	96.64%
F1 score	81.99%	91.22%	93.21%	94.63%	94.40%	95.88%	96.10%	96.04%
TAWA + STD								
Accuracy	82.36%	91.34%	93.32%	94.63%	95.37%	96.15%	96.40%	97.10%
F1 score	83.01%	91.91%	93.61%	94.91%	95.55%	96.18%	96.33%	96.57%

**Table 5:** Performance comparison of pedestrian identification.

Method	Accuracy	Precision	Recall	F-Measure
FF [11] (2 steps)	57.25%	59.30%	56.79%	57.69%
PPAC [12] (2 steps)	88.15%	88.09%	88.15%	88.10%
Proposed (2 steps)	91.34%	91.97%	91.88%	91.91%
PPAC [12] (1 steps)	75.01%	74.57%	75.01%	74.58%
LSTM [15] (1 steps)	76.56%	76.58%	73.38%	74.94%
Proposed (1 steps)	82.36%	83.09%	82.97%	83.01%

## Data Availability

The data are not publicly available due to privacy restriction.
